# Supplementary material for: The genome sequence of the Suspected, Parastichtis suspecta (Hübner, 1809) (Lepidoptera: Noctuidae)
Source: Wellcome Open Res. 2026 May 18;11:288. [Version 1] doi: 10.12688/wellcomeopenres.26587.1 (PMC13276485; doi:10.12688/wellcomeopenres.26587.1)
Supplement: Peer review history

# Reviewer report: Reviewer response for version 1

**DOI:** 10.21956/wellcomeopenres.29280.r156712

**Published:** 2026-06-17

**Authors:** Jonathan Badger

**Affiliations:** 1 Laboratory of Integrative Cancer Immunology (LICI), National Cancer Institute Center for Cancer Research, Bethesda, Maryland, USA; 2 National Cancer Institute, Misrata, Libya

The authors describe a chromosome-level genome assembly of the noctuid moth *Parastichtis suspecta*, which inhabits Britain. The more complete haplotype was scaffolded into 30 pseudomolecules including the Z sex chromosome (which many insect genome projects fail to obtain, so this is good to see). The manuscript is correctly formatted for a data note and the standard metrics such as N50 and BUSCO are reported and look reasonable. I have checked to see if the data is in the ENA as reported and it is.

Are sufficient details of methods and materials provided to allow replication by others?

Yes

Is the rationale for creating the dataset(s) clearly described?

Yes

Are the datasets clearly presented in a useable and accessible format?

Yes

Are the protocols appropriate and is the work technically sound?

Yes

Reviewer Expertise:

I am a computational genomicist. While I primarily work in the biomedical field, I have also worked on several insect genomes as well.

I confirm that I have read this submission and believe that I have an appropriate level of expertise to confirm that it is of an acceptable scientific standard.

# Reviewer report: Reviewer response for version 1

**DOI:** 10.21956/wellcomeopenres.29280.r156714

**Published:** 2026-06-17

**Authors:** Oliver Rupp

**Affiliations:** 1 Justus-Liebig-University, Giessengiessen, Germany

This article presents the assembly of Parastichtis suspecta.

 The data creation and data analyses follow state-of-the-art methods for eukaryotic genome assemblies established at the Darwin Tree of Life Consortium, including PacBio Hifi and Hi-C sequencing. The assembly with hifiasm in Hi-C mode was followed by Hi-C scaffolding and manual curation.

 The assembly quality assessment using k-mer and orthology based methods like Merqury.FK or BUSCO showed a high-quality sequence for both haplotypes.

 The methods are clearly presented, and the quality of the data is shown.

 Overall, no major issues are to report.

Are sufficient details of methods and materials provided to allow replication by others?

Yes

Is the rationale for creating the dataset(s) clearly described?

Yes

Are the datasets clearly presented in a useable and accessible format?

Yes

Are the protocols appropriate and is the work technically sound?

Yes

Reviewer Expertise:

genome assembly

I confirm that I have read this submission and believe that I have an appropriate level of expertise to confirm that it is of an acceptable scientific standard.

# Reviewer report: Reviewer response for version 1

**DOI:** 10.21956/wellcomeopenres.29280.r156713

**Published:** 2026-06-04

**Authors:** Anwesha Das

**Affiliations:** 1 Sanaka Educational Trust’s Group of Institutions (SETGOI), West Bengal, India

This manuscript entitled "The genome sequence of the Suspected, *Parastichtis suspecta* (Hübner, 1809) (Lepidoptera: Noctuidae)" reports a **chromosome-level genome assembly** for *Parastichtis suspecta* (Lepidoptera: Noctuidae), generated as part of the **Darwin Tree of Life (DToL) Project**. The authors sequenced an individual male specimen collected in Hampshire, UK, using **PacBio HiFi long reads** (≈54× coverage) and **Hi-C data** for scaffolding. Key results include:

 Assembly size: ~869 Mb for each haplotype

 Haplotype 1: 99.8% assigned to **30 chromosomal pseudomolecules**, including the Z chromosome

 Scaffold N50: ~29.4 Mb; contig N50 ~9.77 Mb

 BUSCO completeness: ~99% (lepidoptera_odb10)

 Estimated QV: ~69

 Combined haplotype k-mer completeness: ~99.35%. The manuscript provides detailed methodological descriptions, sequencing protocols, assembly pipeline steps, and quality metrics. Data are publicly available via ENA. This work contributes to ongoing efforts to generate **reference genomes for biodiversity research**.

 This is a **high-quality genome resource paper** consistent with DToL “data note” standards. The assembly appears technically robust and meets **Earth BioGenome Project (EBP)** benchmarks (6.C.Q69). However, as a scientific contribution, the manuscript is **limited in biological interpretation and contextualisation**. While this is acceptable for a data note, several aspects require clarification or expansion to ensure **scientific transparency, reproducibility, and utility**.

 Major Strengths

**High assembly quality**

 Chromosome-level scaffolding with strong contiguity and completeness

**Use of modern sequencing approaches**

 PacBio HiFi + Hi-C phasing

**Thorough methodological documentation**

 Clear protocols and software versions

**Data availability**

 Open access raw data and assembly

 Major Concerns

 Lack of Biological Insight or Interpretation

**Issue:**

 The manuscript provides almost no biological interpretation of the genome.

**Why this matters:**

 Even for data notes, minimal discussion of genome features improves scientific value and reuse.

**Required actions:**

 Add a short section addressing:

 Genome size comparison with related Noctuidae species

 Repeat content interpretation (43.76% reported but not discussed)

 Any notable features (e.g., chromosome count consistency with Lepidoptera norms)

 Provide at least 1–2 comparative references to contextualise results

 Incomplete Reporting of Assembly Quality Metrics

 Issue:

 Some key metrics are insufficiently explained or missing:

 No explicit contamination statistics reported despite ASCC use

 No detailed BUSCO lineage justification

 No structural accuracy validation beyond Hi-C visual inspection

 Required actions:

 Add:

 Quantitative contamination results (e.g., % removed, taxonomic origin)

 Clarification of why lepidoptera_odb10 was selected

 Statement on structural accuracy validation (e.g., misjoins detected, correction rationale)

 Limited Explanation of Haplotype Treatment

 Issue:

 Two haplotypes were generated, but the biological relevance and handling are unclear.

 Required actions:

 Clarify:

 Whether haplotypes represent a phased diploid genome or alternative assemblies

 Why is haplotype 2 only scaffold-level

 Recommended version for downstream analysis

 Provide clear guidance for users of the dataset

 K-mer Completeness Interpretation is Confusing

 Issue:

 Individual haplotypes show ~70% completeness vs 99.35% combined

 This is not explained

 Required actions:

 Explain:

 Why haplotype-specific completeness is low

 Whether this reflects heterozygosity (1.69%) or partitioning effects

 Add a brief explanatory paragraph to prevent misinterpretation

 Missing Annotation Information

 Issue:

 Genome annotation is deferred but not described.

 Required actions:

 Clearly state:

 Expected timeline or status of annotation

 Whether RNA-seq data exists

 Where annotation will be hosted (Ensembl mentioned but not elaborated)

 Minor Comments

 Taxonomic Inconsistency

 The species authority differs: “(Hübner, 1809)” vs “(Hübner, 1817)”

 Fix: Ensure consistency throughout

 Typographical / Formatting Issues

 Spacing issues (e.g., “fromOrkney”, “northWales”)

 Figure captions sometimes difficult to parse

 Fix: Proofread carefully

 Figures

 Figures are referenced but not fully interpreted

 Fix: Add brief interpretation (e.g., Hi-C confirms chromosomal structure)

 Sampling Context

 Only one individual was analysed

 Fix: Add statement acknowledging limitations (e.g., population variation not captured)

 Methods Clarity

 Some protocols referenced externally only

 Fix: Summarise key steps inline (without requiring external lookup)

 Aspects Cannot Be Fully Assessed

 Biological accuracy of taxonomy and species identification beyond the provided description

 Raw data integrity (FASTQ/BAM not evaluated)

 Performance of assembly under alternative pipelines

 Functional annotation quality (not included)

Are sufficient details of methods and materials provided to allow replication by others?

Partly

Is the rationale for creating the dataset(s) clearly described?

Partly

Are the datasets clearly presented in a useable and accessible format?

Partly

Are the protocols appropriate and is the work technically sound?

Partly

Reviewer Expertise:

Medicinal chemistry

I confirm that I have read this submission and believe that I have an appropriate level of expertise to confirm that it is of an acceptable scientific standard, however I have significant reservations, as outlined above.

# Reviewer report: Reviewer response for version 1

**DOI:** 10.21956/wellcomeopenres.29280.r155911

**Published:** 2026-06-02

**Authors:** Gulab D Khedkar

**Affiliations:** 1 Dr Babasaheb Ambedkar Marathwada University, Aurangabad, Maharashtra, India

Review report on the manuscript entitled, “The genome sequence of the Suspected, Parastichtis suspecta (Hübner, 1809) (Lepidoptera: Noctuidae)”

 This manuscript presents a high-quality chromosome-level genome assembly of *Parastichtis suspecta*, an ecologically important noctuid moth species and provides a valuable genomic resource for Lepidopteran biology, comparative genomics, biodiversity assessment, and evolutionary studies. The manuscript is well organized, technically rigorous, and clearly written. The authors successfully generated a highly contiguous and well-curated assembly using PacBio HiFi long-read sequencing combined with Hi-C scaffolding. The assembly statistics are excellent, with 99.8% of the assembly assigned to 30 chromosomal pseudomolecules, including the Z chromosome. The reported scaffold N50 (29.41 Mb), high consensus QV values (~69), and BUSCO completeness of 99.0% clearly demonstrate the robustness and reliability of the assembly. The assembly also meets the Earth BioGenome Project reference standards, highlighting the quality of the genomic resource.

 A major strength of the manuscript is the comprehensive methodological transparency. The workflows for specimen collection, DNA barcoding verification, nucleic acid extraction, genome assembly, curation, and quality assessment are described in sufficient detail to ensure reproducibility. The inclusion of openly accessible accession numbers, software versions, and analysis pipelines further enhances the utility of the work for the broader scientific community. The background section provides useful ecological and taxonomic context for *P. suspecta*, including its distribution, habitat associations, and biological characteristics. The authors appropriately emphasize the importance of generating genomic resources for biodiversity documentation and future conservation-oriented studies.

 The manuscript is scientifically sound and technically complete. The discussion could briefly elaborate on how this genome may contribute to future comparative analyses within Noctuidae and broader Lepidopteran phylogenomics. It is a minor and do not detract from the overall scientific quality of the work.

 Overall, the manuscript presents an important and high-quality genomic resource generated using state-of-the-art methodologies. The study will be valuable to researchers working in Lepidoptera genomics, evolutionary biology, biodiversity science, and conservation genomics. I recommend the manuscript for acceptance after minor revision.

Are sufficient details of methods and materials provided to allow replication by others?

Yes

Is the rationale for creating the dataset(s) clearly described?

Yes

Are the datasets clearly presented in a useable and accessible format?

Yes

Are the protocols appropriate and is the work technically sound?

Yes

Reviewer Expertise:

Genomics, biodiversity research

I confirm that I have read this submission and believe that I have an appropriate level of expertise to confirm that it is of an acceptable scientific standard.
